# Calcitonin gene‐related peptide: An intra‐articular therapeutic target for TMJ disorders

**DOI:** 10.1002/cre2.606

**Published:** 2022-06-14

**Authors:** Sabine M. Brouxhon, M. Kerry O'Banion, Ian M. Dickerson, Stephanos Kyrkanides

**Affiliations:** ^1^ Department of Physiology, School of Medicine Stony Brook University Stony Brook New York USA; ^2^ Department of Neuroscience, The Del Monte Institute for Neuroscience University of Rochester School of Medicine & Dentistry Rochester New York USA; ^3^ Department of Oral Health Science, College of Dentistry University of Kentucky, and Department of Neurology, University of Rochester School of Medicine & Dentistry Rochester New York USA

**Keywords:** CGRP, gene therapy, osteoarthritis, TMJ disorders

## Abstract

**Objectives:**

The goal of this project was to evaluate the role of calcitonin gene‐related peptide (CGRP) in the development of arthritis.

**Methods:**

Herein, we employed somatic mosaic analysis in two different joints by FIV(CGRP) intra‐articular inoculation in the knees or temporomandibular joints (TMJ) of young adult male C57/BL6 mice. FIV(CGRP) is a feline immunodeficiency virus over‐expressing full‐length CGRP. Joint pathology and function were evaluated at the histopathological and behavioral levels. In addition, CGRP signaling was inhibited by intra‐articular inoculation using FIV(CGRP_8‐37_), such that the inhibitory peptide CGRP(8‐37) was overexpressed 4 weeks after induction of joint inflammation in the TMJ of IL‐1β^XAT^ transgenic mouse model. The mice were evaluated for behavior and killed for evaluation of knee and TMJ pathology.

**Results:**

Overexpression of CGRP in the joints of wild‐type mice induced the development of joint anomalies, including meniscal hypertrophy and articular pathology, associated with nocifensive behavior. Intriguingly, overexpression of the CGRP(8‐37) inhibitory peptide in the knee and TMJ of IL‐1β^XAT^ transgenic mice with joint inflammation resulted in partial amelioration of the attendant joint pathology.

**Conclusions:**

The results of this study suggest that CGRP is sufficient and necessary for the development of joint pathology and may serve as an intra‐articular therapeutic target using gene therapy or monoclonal antibody‐based therapies.

## 1 INTRODUCTION

Calcitonin gene‐related peptide (CGRP), a 37 amino acid neuropeptide found in small diameter fibers, acts through its cognate receptor calcitonin receptor‐like receptor (CLR) that is linked to an essential receptor activity modifying protein (RAMP) necessary for functionality (Dickerson, 2013). CGRP is primarily produced by thinly myelinated Aδ and unmyelinated C nerve sensory nerve fibers and plays a key role in peripheral sensitization and induction of pain‐related pathways. In addition, CGRP is a potent vasodilator involved in migraine physiology, whereby monoclonal antibodies against CGRP and its receptor have recently been FDA‐approved for the treatment of migraines (Russell et al., 2014). CGRP can further activate and sensitize trigeminal primary afferent neurons (Romero‐Reyes et al., 2015). To this end, work by our group and others has shown the involvement of increased CGRP levels in animal models of joint disorders (Kido et al., 1993; Lai et al., 2006). In addition, Romero‐Reyes and colleagues (2015) administered the small molecule MK8825, a selective CGRP receptor antagonist, in a mouse model of acute orofacial masseteric muscle pain that was employed as a surrogate of acute temporomandibular joint disorders. Here, mice pretreated with MK8825 showed alleviated orofacial pain behaviors and reduced neuronal activation in the trigeminal nucleus in response to complete Freund's Adjuvant into the masseter muscle. Furthermore, CGRP is elevated in human TMJ specimens harvested from patients with TMJ disorders and increased in the TMJ of individuals with an open bite (Alstergren et al., 1995; Cady et al., 2011; Haeuchi et al., 1999; Kopp, 2001; Romero‐Reyes et al., 2015; Sato et al., 2004). Taken together, the aforementioned studies demonstrate that CGRP is involved in pain physiology, suggesting that therapeutic modalities targeting CGRP may translate into effective therapeutic strategies for painful joint‐related disorders.

We previously demonstrated the role of neuroinflammation in TMJ disorders. Specifically, Fiorentino and colleagues (Fiorentino et al., 2008) demonstrated that centrally induced neuroinflammation involving CGRP in the TMJ contributed to marked histopathological changes of the articular cartilage consistent with initial stages of osteoarthritis. In these studies, inhibition of central neuroinflammation restrained the development of articular pathology in a mouse model of TMJ osteoarthritis (Fiorentino et al., 2008; Lai et al., 2006). Lastly, Kyrkanides et al. (2007) demonstrated that inhibition of afferent sensory signals from the TMJ ameliorated the attendant articular pathology in the mouse model of TMJ inflammation (Lai et al., 2006). Taken together, these studies demonstrate that neuroinflammation in the TMJ is sufficient and necessary for the development of articular pathology in the mouse.

Although the literature suggests that CGRP may contribute to the development of joint pathology, there is a lack of direct proof for the role of CGRP in arthritis. Therefore, the goal of this preclinical study was to elucidate whether CGRP is necessary or sufficient for the development of joint pathology. This is significant, due to the recent FDA approval of monoclonal antibody‐based therapies against CGRP for the management of migraines, which could seamlessly segue into a drug‐repositioning agent for the management of pain‐related osteoarthritic joint disorders.

## 2 MATERIALS AND METHODS

### 2.1 Vector construction and packaging

**Figure 1:**
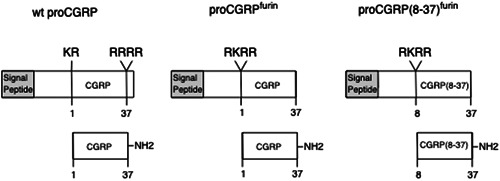
cDNA constructs used for expression of full‐length CGRP and CGRP(8‐37) antagonist. Wild‐type CGRP is expressed as a propeptide, which after removal of its signal peptide undergoes two posttranslational endo‐proteolytic events cleaved by prohormone convertase 1 (PC1) at paired basic amino acids (KR), and furin at tetrabasic sites (RRRR or RKRR). The carboxyl amino acid is glycine, which is converted to a carboxyl amide. ProCGRP^furin^ replaces the neuroendocrine‐specific PC1 cleavage site with a more frequent furin cleavage site and eliminates the propeptide downstream of the carboxyl glycine. ProCGRP(8‐37)^furin^ is processed similarly and has had the first 7 aa of mature CGRP deleted. Numbering refers to amino acid positions in the mature CGRP molecule. cDNA, complementary DNA; CGRP, calcitonin gene‐related peptide.

The FIV(CGRP) and FIV(CGRP8‐37) transfer vectors expressing full‐length CGRP and the inhibitory peptide CGRP(8‐37), respectively, were constructed as follows. Two plasmids were constructed that contained the rat proCGRP complementary DNA (cDNA), modified to express either wild‐type CGRP or the CGRP(8‐37) antagonist, which had the first 7 aa deleted by polymerase chain reaction (PCR) using the primer sets described in Table 1. CGRP is expressed as a propeptide that must undergo two proteolytic cleavages followed by carboxyl amidation to achieve bioactivity (Rosenblatt & Dickerson, 1997). To simplify expression, the prohormone convertase‐1 (PC‐1) site in both constructs was replaced at the NH2‐terminus of CGRP with a furin cleavage site to facilitate correct processing in a wide range of tissues (Seidah & Prat, 2012). At the carboxyl end of CGRP, 8 aa are usually removed by a second cleavage event to expose a carboxyl glycine, which is a substrate for subsequent amidation. Amplification of both constructs ended at this carboxyl glycine, eliminating the need for the downstream cleavage event. Thus, this construct required a single posttranslational furin cleavage to produce the functional peptides. The cDNA for rat proCGRP was cloned into the plasmid pBluescript and used as a template for two overlapping PCR reactions. The first PCR reaction amplified the NH2‐terminal half of the proCGRP molecule, including signal peptide and propeptide sequence, with the downstream primer encoding changes that replaced the PC1 cleavage site (KR) with the furin cleavage site (RKRR), and in the antagonist, PCR also deleted the first 7 aa of CGRP. The COOH‐PCR used a complementary upstream primer to encode the furin cleavage site and delete the first 7 aa of CGRP, with a downstream primer that ended amplification at the carboxyl glycine. The two PCR reactions were purified, denatured, annealed, and used as a substrate for a third PCR reaction using just the two outside primers, resulting in two amplimers encoding the cDNA for either full‐length proCGRP or proCGRP(8‐37), which will produce the antagonist CGRP(8‐37) when introduced into cells (Figure 1). Additionally, the outside primers encoded *Xba*I (upstream) and *Bam*HI (downstream) restriction sites to facilitate cloning into the expression plasmid pBluescript (Table 1). The two constructs were then subcloned into the *XBa*I—*Pac*I sites of the pcDF1‐mcs2‐EF1‐copGFP FIV backbone vector (SBI). The final constructs were sequenced to verify accuracy.

**Table 1:** The PCR primer sequences used to generate proCGRP8‐37 and full‐length CGRP DNA sequences that were cloned into the cloning site of the viral vector used to inoculate the animals

Gene	Primers		
proCGRP_8‐37_	–NH_2_ end	CGRP.Xba	AGCGCTAGCCGTTCTAGACCGCCACCATGGGCTTTCTGAAGTTCTCC
		CGRP.f2	CAGCCGATGGGTCACGCAGGTGGCAGTGTTGCAGGACCTGCGCTTG
			CGCTGGGCAGTGACACT
	–COO^−^ end	CGRP.Bam	CGTAGCCGTTGAGGATCCCTAGCCGAAGGCTTCAGAGCCCA
		CGRP.f1	AGTGTCACTGCCCAGCGCAAGCGCAGGTCCTGCAACACTGCCACCTG
			CGTGACCCATCGGCTG
proCGRP	–NH_2_ end	CGRP.Xba	AGCGCTAGCCGTTCTAGACCGCCACCATGGGCTTTCTGAAGTTCTCC
		CGRP.f4	TCCCGACCTGCTCAGCAAGCCTGCCAGCCGATGGGTCACCCTGCGCT
			TGCGCTGGGCAGTGACACT
	−COO^−^ end	CGRP.Bam	CGTAGCCGTTGAGGATCCCTAGCCGAAGGCTTCAGAGCCCA
		CGRP.f3	AGTGTCACTGCCCAGCGCAAGCGCAGGGTGACCCATCGGCTGGCAG
			GCTTGCTGAGCAGGTCGGGA

Abbreviations: CGRP, calcitonin gene‐related peptide; PCR, polymerase chain reaction.

The FIV vectors were packaged in 293FT cells (Invitrogen, Waltham, MA) cultured in T75 flasks, which were grown to subconfluency in Dulbecco's modified Eagle's medium plus 10% fetal bovine serum (Gemini, Woodland, CA). The cells were then cotransfected with the FIV transfer vector, the packaging and the VSV‐G pseudotyping vectors using Lipofectamine 2000 reagent (Invitrogen) per manufacturer's instructions. Twenty‐four hours after transfection, the supernatant medium was discarded and replaced by a fresh medium. Sixty hours after transfection, the virus‐rich supernatant was collected, filtered through a 0.45 µm SurfilR‐MF filter (Corning Separations Division; Acton, MA) and subsequently concentrated by overnight centrifugation at 7000 g using a Sorvall RC5B high‐speed centrifuge and an SLA‐3000 rotor. Subsequently, the supernatant was decanted, and the viral pellet was resuspended overnight in 1 ml of normal buffered saline containing 40 mg/ml lactose at 4°C. The viral solution was then aliquoted and frozen (−80°C) until further use. Viral vector titers were established using CrfK cells (American Tissue Culture Collection, Manassas, VA) cultured in 24‐well tissue culture plates. Because the FIV backbone vector carries the reporter gene *gfp*, titers were calculated based on the number of gfp–positive cells counted under a fluorescent microscope and extrapolated based on the dilution factor. Titers routinely ranged between 10^7^ and 10^8^ infectious particles/ml.

### 2.2 Animal studies

All animal procedures described were reviewed and approved by the University of Rochester Institutional Animal Care and Use Committee (University Committee on Animal Resources) for compliance with federal regulations before the initiation of the study (OLAW/PHS Assurance A3292‐01). All mice were maintained in an AAALAC‐accredited specific pathogen‐free barrier facility. All procedures followed the AVMA guide per institutional policy. All mice were males and they were housed with five animals per cage. The mice were routinely anesthetized via intraperitoneal injection of ketamine (40 mg/kg). The mice did not experience any adverse events.

The ARRIVE guidelines for animal research were followed. Specifically, this was a comparative study between CGRP‐treated, CGRP8‐37‐treated, and control (gfp‐treated) mice. The sample size is mentioned below. All male mice in the litter were included and there were no exclusion criteria. The mice were randomized into various groups. The operators that handled the mice were not aware of the mice groupings. The outcome measures were again collected blinded to the mice groupings. The statistical method used is described in detail below. Details on the experimental animals and procedures are detailed below. The results are described in detail below.

Pertaining to CGRP overexpression in the knees, 8‐week‐old wild‐type C57BL/6 mice (*N* = 10) were randomly assigned to receive either FIV(CGRP). An additional group (*N* = 6) was randomly assigned to revive FIV(gfp) and served as controls. All mice were anesthetized with ketamine (40 mg/kg), and a 10 µl aqueous solution containing a total of 10^5^ FIV(CGRP) infectious particles was injected into the right and left knee joints of the hind limbs. The knee area was located by palpation. A 27.5G needle was inserted into the joint space from a lateral approach and viral vector solution was injected intra‐articularly. After injection, the mice were returned to their cages and monitored for full recovery from anesthesia. No inclusion/exclusion criteria were applied for these animals. The operator was only aware of the animals’ group assignments.

For the experiments pertaining to CGRP overexpression in the TMJs, 3‐month‐old wild‐type C57BL/6 mice (*N* = 5) were randomly assigned to receive 10 µl containing 10^5^ FIV(CGRP) infectious particles in the right and left TMJ under the surgical plane of anesthesia and returned to their cages. Mouse behavior was subsequently evaluated when the mice were killed 8 weeks later, and their TMJ histology was compared to that of wild‐type controls (*N* = 5). No inclusion/exclusion criteria were applied for these animals. JHM was only aware of animals’ group assignments.

For the experiments pertaining to CGRP(8‐37) overexpression in the TMJs, 3‐month‐old Col1‐IL‐1β^XAT^ mice (Lai et al., 2006) (*N* = 4) were injected with 10 µl containing 1 × 10^6^ FIV(Cre) infectious particles in the right and left TMJ under the surgical plane of anesthesia as described above. After 4 weeks, the mice also received under the surgical plane of anesthesia a second injection into their right and left TMJ of 10 µl FIV(CGRP_8‐37_). No inclusion/exclusion criteria were applied for these animals. JHM was only aware of animals’ group assignments.

Knee joint function was evaluated by locomotion performance on a rotarod, measuring the time a mouse spends on a rotating cylinder at a constant speed of 20 rpm on the rotarod (Columbus Instruments; Columbus OH). The maximum time on the rotarod was limited to 120 s. No animals were excluded from this evaluation. We did not control for any cofounders.

TMJ function was evaluated by resistance to jaw opening based on the principles of the Pain Adaptation Model, which suggests that pain reduces muscle force (Kyrkanides et al., 2007). These data were compared to data collected during a previous experiment derived from 7 Col1‐IL‐1β^XAT^ transgenic mice were injected with a control FIV vector and 8 Col1‐IL‐1β^XAT^ transgenic mice that received FIV(Cre) injections to activate the joint inflammation process in the TMJ (Kyrkanides et al., 2007). No animals were excluded from this evaluation. We did not control for any cofounding conditions as there were none.

At the end of each experiment, the mice were killed via intraperitoneal injection of pentobarbital (100 mg/kg) followed by decapitation.

The animal data are available upon request.

### 2.3 Histological—Immunohistochemical (IHC) studies

Following fixation in 10% formalin, the mouse heads were dissected, defleshed, and decalcified by immersion in an ethylenediaminetetraacetic acid solution for 7 days at 4°C under constant agitation. The TMJs were then processed with an RHS‐1 microwave tissue processor, after which the samples were embedded in paraffin, cut on a microtome as 3 µm thick sections, and collected on glass slides. Overall TMJ histopathology was evaluated in sections stained by Alcian blue‐orange G and Safarin‐O histochemistry (Lai et al., 2006).

IHC analysis was performed as previously described (Lai et al., 2006) using the polyclonal antibody MU33 (dilution 1:1000) raised against the amidated‐CGRP ligand (Rosenblatt & Dickerson, 1997) and with polyclonal antibody NY1021 (dilution 1:1000) raised against CLR, as described previously (Supowit et al., 2011). The histology sections were evaluated under light microscopy using an Olympus BX51 microscope. Microphotographs were captured using a Spot CCD digital camera (Diagnostic Imaging, Sterling Heights, MI) attached to the microscope.

### 2.4 Statistical analysis

Analysis of rotarod data was performed with a repeated measure two‐way analysis of variance with Tukey's Multiple Comparisons (GraphPad Prism, v. 9.1, San Diego, CA). An adjusted *p* < .05 was set as significant.

## 3 RESULTS

### 3.1 Wild‐type mice display normal TMJ cytoarchitecture

**Figure 2:**
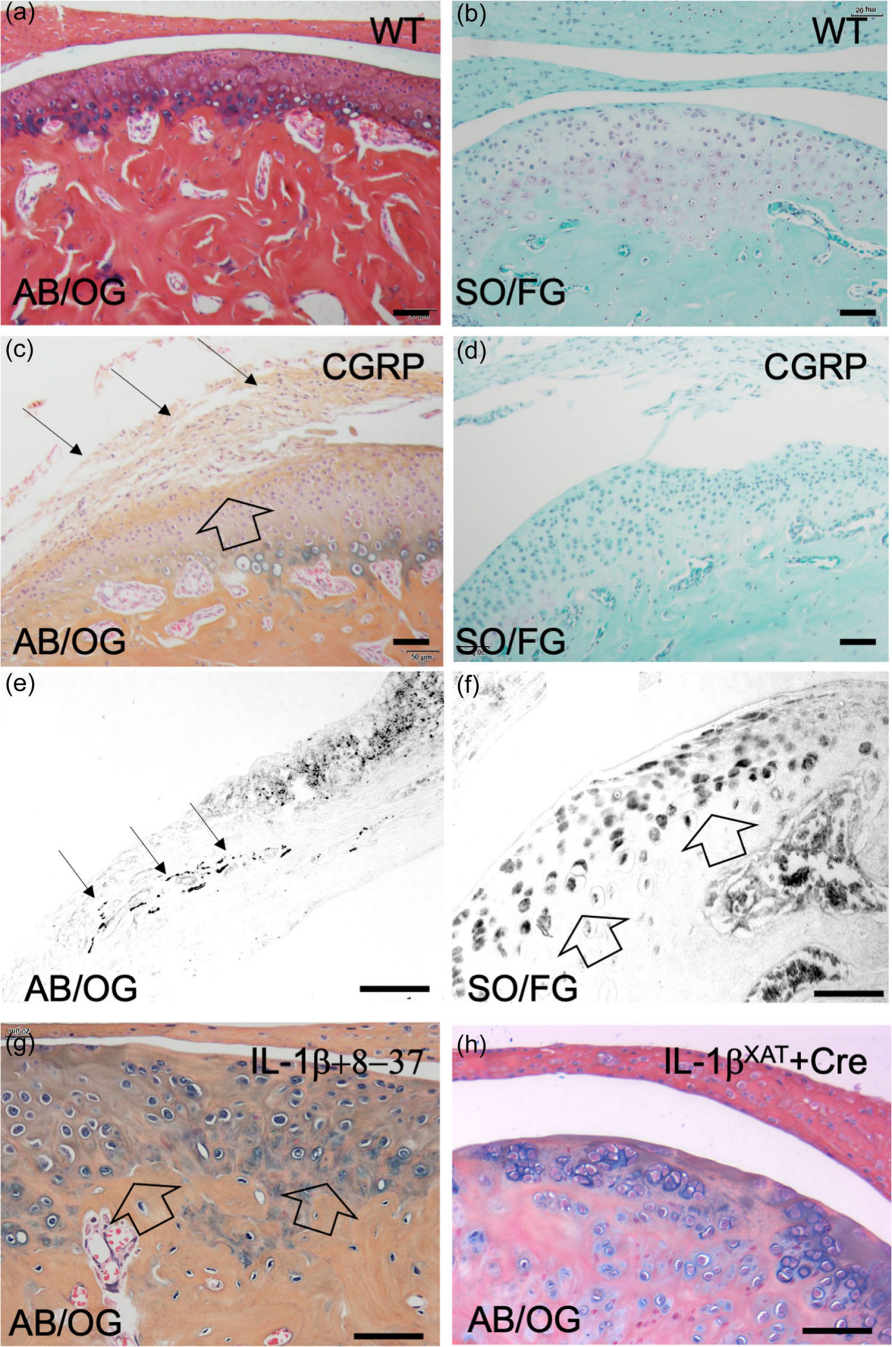
CGRP is sufficient and necessary for the development of TMJ pathology. Representative histological images of TMJ specimens from young adult wild‐type mice depict (a) normal cytoarchitecture of the condylar articular chondrocytes and joint meniscus harvested from wild‐type mice as detected by Alcian‐Blue/Orange‐G histochemistry (AB/OG). (b) The presence of proteoglycans (purple stain) in the wild‐type mouse TMJ is detected by Safarin‐O histochemistry (SO/FG). (c) Histopathological changes in TMJ articular cartilage (open arrow) and meniscus (closed arrows) resulting from overexpression of the full‐length CGRP following intra‐articular inoculation of FIV(CGRP). (d) The absence of proteoglycans in the TMJ after FIV(CGRP) inoculation (absence of purple staining) compared to control in panel B is noted. (e) Representative histological images of CGRP immunohistochemistry and its cognate receptor in mice with TMJ inflammation. Expression of CGRP in the TMJ meniscus (closed arrows) and (f) CGRP (CLR) receptor expression (open arrows) in articular chondrocytes. (g) Intra‐articular overexpression of the inhibitory peptide CGRP(8‐37) partially alleviated the attendant articular pathology in the TMJ of Col1‐IL1β^XAT^ transgenic mice suffering from TMJ inflammation (Lai et al., 2006) (h). CGRP, calcitonin gene‐related peptide; TMJ, temporomandibular joints.

Wild‐type mice with naïve TMJs display normal cytoarchitecture as stained by Alcian blue‐orange G histochemistry (Figure 2a), whereby healthy cartilage (blue stain) comprised of the most superficial resting cell layer, then the proliferative chondrocyte layer, followed by the hypertrophic chondrocyte zone. The articular cartilage is supported by bone (red stain). Proteoglycans are evidenced throughout the articular cartilage by Safarin‐O histochemistry (Figure 2b) detected by a purple stain on a green background.

### 3.2 Overexpression of CGRP in the mouse TMJ resulted in the development of joint pathology

Eight weeks following inoculation of the TMJ with intra‐articular injections of FIV(CGRP), we observed the development of a hypertrophic meniscus (closed arrows) along with thickening and erosion (open arrow) of the articular cartilage (Figure 2c) compared to controls (Figure 2a). Simultaneously, we observed a decrease in proteoglycan content in the articular cartilage as evidenced by the lack of Safarin‐O staining (Figure 2d) compared to controls (Figure 2b).

### 3.3 The CGRP and its cognate receptor are present in the TMJ

CGRP was detected by immunohistochemistry in nerve bundles located in the retrodiscal tissue (closed arrows) of the TMJ meniscus (Figure 2e—black stain). The CGRP receptor was localized by immunohistochemistry using an antibody against CLR in TMJ articular chondrocytes (Figure 2f—black stain).

### 3.4 The CGRP(8‐37) antagonist ameliorated attendant joint pathology in mice with TMJ inflammation

Intra‐articular overexpression of the inhibitory peptide CGRP(8‐37) after inoculation of the TMJ with the pertinent FIV vector partially ameliorated the attendant joint pathology observed in the inflamed TMJ (Figure 2g), including restoration of the articular cyto‐architecture (open arrow) compared to osteoarthritic TMJ (Figure 2h).

### 3.5 CGRP overexpression in the TMJ results in nocifensive behavior in mice

Eight weeks following inoculation of the TMJ with FIV(CGRP), we observed the development of intra‐articular overexpression of CGRP in the TMJ resulted in a reduction of resistance to mouth opening (Table 2) when compared to previously published wild‐type mice values (Figure 2b in Kyrkanides et al., 2007). Specifically, the average value of resistance to mouth opening in the CGRP‐overexpressing mice was 1.712 µNT (SD = 0.38), compared to 3.22 µNT (SD = 0.22) for wild‐type mice. Interestingly, the CGRP‐overexpressing mice's average value was similar to that of mice suffering from TMJ inflammation with 1.74 µNT (SD = 0.23) (Kyrkanides et al., 2007).

**Table 2:** Resistance to mouth opening (Kyrkanides et al., 2007) raw data was recorded from mice that received intra‐articular administration of FIV(CGRP) into the TMJ

No.	Agent	μNT									
46	CGRP	1.7	1.6	1.7	1.7	1.6	1.6	1.6	1.8	1.7	1.6
		1.7	1.5	1.9	1.7	1.8	2.0	1.5	1.9	1.6	1.4
		1.2	1.7	1.6	1.1	1.1	1.1	1.1	1.3	1.3	1.2
		1.7	1.5	1.5	1.2	1.3	1.2	1.1	1.7	1.6	2.0
		1.3	1.3	1.3	1.3	1.5	2.3	1.3	1.4	1.4	1.3
48	CGRP	1.6	1.4	1.9	1.5	1.4	2.1	1.5	1.5	1.6	1.6
		1.9	2.2	2.0	2.1	2.0	1.9	1.8	1.9	2	2.5
		2.4	1.9	2.1	1.9	2.0	2.0	2.0	2.1	2.0	2.0
49	CGRP	2.5	2.0	2.1	2.4	2.7	2.1	2.0	2.2	2.4	2.1
		1.6	1.6	1.9	1.6	1.8	1.8	2.0	1.8	2.4	1.9
		1.8	2.4	2.3	1.8	1.8	1.6	1.8	2.1	2	1.7
		1.8	2.0	2.4	2.3	1.7	1.8	1.7	2.1	1.7	2.0
		1.5	2.0	1.4	1.6	1.6	1.4	1.6	1.5	1.6	1.9
50	CGRP	1.7	1.7	1.9	1.8	1.9	2.3	1.7	1.8	1.9	2.4
		1.9	2.0	2.0	2.0	2.0	2.1	2.0	2.0	2.0	2.0
52	CGRP	1.4	1.4	1.4	1.4	1.4	1.6	1.5	1.4	1.3	1.8
		1.3	1.1	1.1	1.1	1.2	1.3	1.1	1.3	1.1	1.3
		1.2	1.3	2	1	1.1	1.1	1.1	1.1	1.1	1.2
		1.6	1.9	1.7	1.7	1.6	1.2	1.6	1.7	2.0	1.7
		1.2	1.2	1.4	2.0	1.0	1.1	1.3	0.7	0.7	0.6

*Note*: These data were compared with previously recorded data from mice suffering from TMJ inflammation as well as controls (in the Results section).

Abbreviations: CGRP, calcitonin gene‐related peptide; TMJ, temporomandibular joints.

### 3.6 Overexpression of CGRP in the mouse knee resulted in join pathology and nocifensive behavior

**Figure 3:**
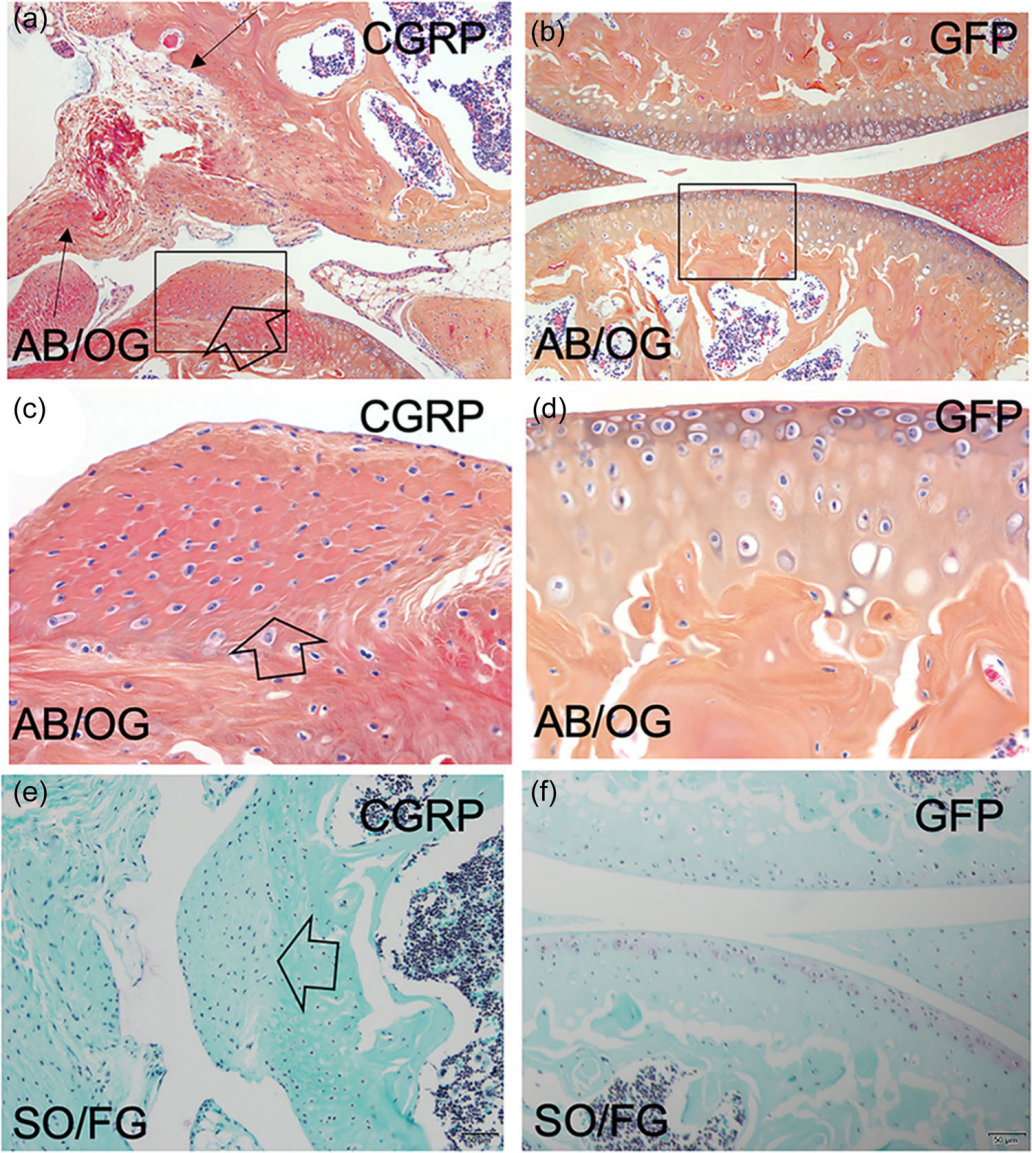
CGRP overexpression in the knee joint induces joint pathology. The role of CGRPP in joints was explored in a somatic mosaic analysis model in the knee joints of wild‐type mice by intra‐articular inoculation with FIV(CGRP). (a) Intra‐articular CGRP overexpression resulted in the development of articular spurring (open arrows), synovial hyperplasia, and soft tissue hypertrophy (closed arrows) compared to (b) controls. (c) Higher magnification of the enclosed area identified in panel A depicts the attendant articular spurring. (d) Higher magnification of the enclosed area identified in panel B depicts normal articular cytoarchitecture. (e) The absence of proteoglycans in the TMJ after FIV(CGRP) inoculation (absence of purple staining) is noted compared to (f) controls. Alcian‐Blue/Orange‐G histochemistry, (AB/OG); Safarin‐O histochemistry, (SO/FG). CGRP, calcitonin gene‐related peptide.

**Figure 4:**
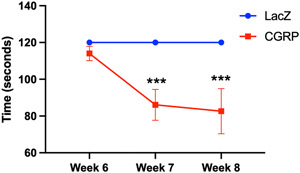
Intra‐articular CGRP overexpression in the knee joints induces nocifensive behavior. Changes in locomotion, as a measure of nocifensive behavior, were evaluated by rotarod in mice inoculated with FIV(CGRP) versus FIV(gfp). Eight weeks following intra‐articular inoculation, mice over‐expressing CGRP in their knee joints showed a significant decline in locomotion, which persisted at 8 weeks in the CGRP group. ***p < .001. CGRP, calcitonin gene‐related peptide.

Eight weeks following inoculation of the TMJ with FIV(CGRP), we observed the development of significant articular spurring, synovial hyperplasia, and soft tissue hypertrophy compared to controls (Figure 3a–d). Qualitative analysis of proteoglycan content by Safranin‐O histochemistry showed depletion of proteoglycan content compared to controls (Figure 3e,f). Moreover, CGRP overexpression in the knee joints also resulted in significant nocifensive behavior, as detected by rotarod analysis (Figure 4).

## 4 DISCUSSION

The goal of this preclinical study was to elucidate whether CGRP is necessary or sufficient for the development of articular pathology in mice. To this end, we used viral delivery to acutely increase CGRP expression in the mouse knee joints or TMJ. Our results demonstrate that intra‐articular CGRP overexpression is sufficient in the development of meniscal hypertrophy, with loss of proteoglycans in the articular cartilage and spurring in joints. In addition, intra‐articular CGRP overexpression resulted in the development of nocifensive behavior, as detected by decreased time spent on a rotarod. These results are in agreement with previous studies where we observed decreased articular pathology following competitive inhibition of CGRP signaling in the Col1‐IL1β^XAT^ mouse model of TMJ inflammation (Lai et al., 2006).

The potential role of CGRP in joint disorders has been previously suggested by a number of descriptive studies analyzing specimens harvested from human patients, as well as small laboratory animal models (Alstergren et al., 1995; Bullock et al., 2014; Cady et al., 2011; Haeuchi et al., 1999; Sato et al., 2004). However, this is the first study to demonstrate that CGRP is sufficient to induce articular pathology in the mouse. Moreover, our results build on the evidence produced by Romero‐Romero‐Reyes et al. (2015) whereby the small molecule MK8825, a selective CGRP receptor antagonist, alleviated orofacial pain behaviors, and reduced neuronal activation in the trigeminal nucleus in response to complete Freund's Adjuvant into the masseter muscle.

Benschop et al. (2014) generated a neutralizing antibody to CGRP, namely LY2951742, which was tested in preclinical in vivo models of osteoarthritis pain in the rat. Neutralization of CGRP significantly reduced pain behavior as measured by a weight‐bearing differential in the rat mono‐iodoacetate model in a dose‐dependent manner. In addition, pain reduction after CGRP neutralization was independent of prostaglandins. Importantly, neutralization of CGRP also provided dose‐dependent and prolonged (>60 days) pain reduction in the rat meniscal tear model of osteoarthritis after only a single injection of LY2951742. However, when this antibody was administered subcutaneously to patients suffering from moderate to the severe knee joint pain due to osteoarthritis, as part of a double‐blind, double‐dummy, placebo, and active‐controlled phase‐II clinical trial, it failed to produce analgesia after 4 months of treatment (Jin et al., 2016). The trial was consequently discontinued due to inadequate efficacy. We believe that this is a result of the following limitation: The administration of LY2951742 to patients was subcutaneous, versus intra‐articular, and therefore exposed the antibody to host defense mechanisms that likely neutralized its function. Moreover, as our results show, the effect of CGRP is intra‐articular in nature; conversely, subcutaneously administered antibodies will have limited access to intra‐articular tissues due to the presence of the joint capsule.

Taken together, our results described herein and together with the available literature, lend to the development of a model whereby continuous peripheral injury and/or inflammation in the TMJ will result, over time, in the antidromic stimulation and release of CGRP by small diameter C and Aδ fibers through a sensory dorsal root reflex. In turn, CGRP will have a direct effect on the articular chondrocytes and meniscus, thereby contributing to the development of knee joint or TMJ pathology. Based on our results, we conclude that inhibition of CGRP signaling in the TMJ via intra‐articular administration of antibodies, or other small molecule inhibitors, has the potential to provide lasting alleviation against tissue pathology and attendant symptomatology, especially since inflamed joints are susceptible to joint loading and imbalanced (right‐left) function (Piancino et al., 2015).

## AUTHOR CONTRIBUTIONS

Sabine M. Brouxhon designed the experiments, analyzed the data, and composed the manuscript. M. Kerry O'Banion contributed to the development of the Col1‐IL1betaXAT mouse model, analyzed the data, and composed the manuscript. Ian M. Dickerson developed the DNA fragments for overexpressing CGRP and CGRP8‐37, analyzed the data, and composed the manuscript. Stephanos Kyrkanides designed and carried out the experiments, collected and analyzed the data, and composed the manuscript.

## CONFLICT OF INTEREST

The authors declare no conflict of interest.

## Data Availability

The data sets used and analyzed during the current study are available from the corresponding author on reasonable request.
